# Genome-wide association analysis of HDL-C in a Lebanese cohort

**DOI:** 10.1371/journal.pone.0218443

**Published:** 2019-06-18

**Authors:** Rebecca Deek, Jason Nasser, Anthony Ghanem, Marc Mardelli, Georges Khazen, Angelique K. Salloum, Antoine Abchee, Michella Ghassibe-Sabbagh, Pierre Zalloua

**Affiliations:** 1 Department of Biostatistics, Epidemiology, and Informatics, Perelman School of Medicine, University of Pennsylvania, Philadelphia, PA, United States of America; 2 School of Medicine, Lebanese American University, Beirut, Lebanon; 3 Department of Natural Sciences, School of Arts and Sciences, Lebanese American University, Beirut, Lebanon; 4 Department of Internal Medicine, American University of Beirut, Beirut, Lebanon; 5 Harvard School of Public Health, Boston, MA, United States of America; Università degli Studi di Milano, ITALY

## Abstract

Low serum levels of high-density lipoprotein cholesterol (HDL-C) have been shown to be a risk factor for coronary artery disease independent of low-density lipoprotein cholesterol (LDL-C) in different populations. In this study, we investigated genetic variants through genome-wide association studies to determine their association with HDL-C levels in a sample of 2,700 patients. We identified several SNPs associated with HDL-C levels in the Lebanese population using unadjusted and adjusted by biological factors models. We replicated the association of rs3764261 within *CETP* with HDL-C levels in the study population, and found other previously unidentified SNPs to be significant at the suggestive level, in both previously identified and unidentified genes. This paper reports the first genome-wide analysis of HDL-C in the Lebanese, Middle Eastern, population and supports the importance of genome-wide association studies across different and minor ethnicities to understand better the etiology of complex human diseases.

## Introduction

High-density lipoprotein (HDL) cholesterol has been one of the most widely used factors in cardiovascular risk assessment since the first reports of its strong inverse association with coronary heart disease in large epidemiologic studies [1–3]. HDL is a heterogeneous set of macromolecules composed of quantitatively and qualitatively varying lipids, proteins, and apolipoproteins [4]. The different subpopulations of HDL are formed by the interconversion of lipids by cholesteryl ester transfer protein (CETP), lecithin:cholesterol acyltransferase (LCAT), phospholipid transfer protein (PLTP), endothelial lipase (EL), hepatic lipase (HL), and scavenger receptor BI (SR-BI). The mechanism by which HDL exerts its cardio-protective effects has been hypothesized and evidenced to include anti-inflammatory, anti-oxidative, and endothelial effects; however, its best recognized function is in reverse cholesterol transport which most contributes towards its anti-atherosclerotic protective properties [5, 6]. Atherosclerotic plaque development is central in the pathogenesis of cardiovascular disease and the hallmark of atherosclerotic streaks are lipid laden macrophages known as foam cells, which can accumulate cellular cholesterol in an unregulated manner through scavenger receptors [7]. HDL is thought to mediate the clearance of cholesterol from the periphery, increasing its efflux and working against atherosclerotic plaque build-up, thus actively protecting against cardiovascular disease [8–11]. Serum HDL cholesterol (HDL-C) levels are thus commonly used clinically in cardiovascular risk assessment formulas and are recognized as one of the constituents of the metabolic syndrome.

The epidemiologic evidence linking the measured serum HDL-C to cardiovascular disease risk has been robust [12–14]. Low levels of HDL-C have been shown to be associated with heart disease independent of low-density lipoprotein cholesterol (LDL-C), such that a 1% decrease in HDL-C is associated with a 3–4% increase in coronary artery disease (CAD) over six years [12, 15, 16]. This component of HDL however has not been directly shown to mediate protective effects of HDL, causing its role to be called into question. Arguments against its risk-modulating role come from studies of monogenic disorders in HDL metabolism whereby many Mendelian randomization studies did not show a correlation between HDL-C levels and cardiovascular disease outcomes [17]. Of note however, monogenic disorders leading to decreased CETP function have been consistently associated with increased HDL-C levels and reduced cardiovascular disease risk [18–20].

The National Cholesterol Education Program by the National Lung, Heart, and Blood Institute put forth a report stating that normal HDL-C levels range between 40 mg/dl and 60 mg/dl [21]. Studies have shown that lipid levels vary with respect to ethnicity [22]. The mean estimated HDL-C levels of Lebanese patients undergoing cardiac catheterization is 40–44 mg/dL [23, 24]. The estimated HDL-C levels of the overall Lebanese population is 40.14 ± 0.77 mg/dL for men and 51.67 ± 0.77 mg/dL for women [25]. However, the genetic impact of HDL-C levels on the Lebanese population has not been investigated.

Most dyslipidemia treatments aim to lower LDL-C to reduce the risk of CAD, though there is a small, but growing, focus on treatments that instead alter HDL-C levels [13]. While treatments aiming to modify HDL-C levels via medications such as CETP inhibitors have been in development, results have been disappointing [26]. Despite achieving pharmacologically increased HDL-C levels, a decreased cardiovascular risk has not followed, likely due to pharmacological dissociation between HDL-C levels and HDL functionality [27]. Five genes accounted for nearly 40% of the variation in HDL-C in the European. These are *LIPC*, *CETP*, *ABCA1*, *LPL*, and *LDLR*, with *LIPC* accounting for most of the variation (53%) and *LPL* and *LDLR* accounting for the least (6% each) [28]. *CETP* has been the gene most consistently and strongly associated with HDL-C levels across several studies [29–31].

Genome-wide association studies (GWAS) aim to identify genetic risk factors associated with both common and rare diseases by using common SNPs in the human DNA sequence [32]. A common difficulty facing GWAS is the matter of population substructure. Phenotypes, as well as allelic frequencies, are known to vary with ethnicity, potentially hindering the generalizability of GWAS. Moreover, linkage patterns between sources of genetic variation differ, as expected, because different ethnicities have not had the same amount of time to undergo genetic recombination. However, this variation in linkage disequilibrium may yield a benefit in locating true causal variants [32, 33]. This study is conducted on a previously collected population of CAD cases and controls. The specific aim of this study is to determine which genetic loci are associated with HDL-C levels through genome-wide association analysis, in a population where such studies have not been reported on previously or characterized in the literature.

## Materials and methods

### Study design and data collection

The samples utilized in this study were derived from two separate patient collections. The first included a total of 7,710 Lebanese patients undergoing cardiac catheterization that were enrolled as part of two cross-sectional studies of the FGENTCARD Consortium [34]. While recruiting subjects (cases and controls) from a catheterization laboratory may have some drawbacks, yet it provides the advantage of having a clearly defined phenotype that makes the distinction between cases and controls more robust. These patients were recruited at two major hospitals, the Rafic Hariri University Hospital in Beirut (The Lebanese Capital), and the “Centre Hospitalier du Nord” in north Lebanon, between May 2007 and June 2010. All participants provided written informed consent prior to filling out the questionnaire or giving blood samples. Research and data collection in both cohorts were carried out in compliance with the Helsinki Declaration, with the approval of the LAU Institutional Review Board and the Committee on Human Subjects in Research (CHSR). Catheterization by Judkins’ technique as well as angiography of major vessels were as described elsewhere [34]. Coronary angiograms were reviewed by two interventional cardiologists blinded to the patients’ HDL-C results. The extent of coronary lesion was estimated visually by comparing the reduction in the diameter of the narrowed vessel to a proximal assumed normal arterial segment. Clinical data was retrieved from patients via a questionnaire or hospital records. Blood samples were collected for DNA analysis and/or metabolic analyses.

The second included 775 patients who were enrolled via a cross sectional study of Lebanese Type II Diabetes patients [35]. This study had two sites of enrollment, one in Beirut, and the other in north Lebanon. Clinical data was retrieved from patients via a questionnaire. Blood samples were collected for HbA1C, fasting blood glucose, and lipid profile measurements after 12 hours of fasting and DNA analysis.

Selection criteria were based on the complete availability of the patients’ relevant parameters described in Table 1 as well as the availability of genotype data. Subjects on whom no HDL-C values were obtained were excluded from the analyses.

**Table 1:** Descriptive statistics of the population.

		Mean±SD		Number (%)
**Age**		62.32±11.01		
**Sex**	Male			1965 (72.67%)
	Female			739 (27.33%)
**Diabetes**	Controls			1621 (59.95%)
	Affected			1083 (40.05%)
**CAD**	No stenosis			501 (18.53%)
	≤ 50% stenosis			280 (10.36%)
	>50% stenosis			1923 (71.11%)
**Cholesterol (mg/dL)**		182.89±48.03		
**Triglycerides (mg/dL)**		183.36±112.982		
** LDL-C (mg/dL)**		110.19±40.93		
**WBC**		8.43±2.81		
**Hematocrit**		40.57±4.65		
**BMI**		28.16±4.43		

All participants provided informed consent prior to filling out the questionnaire or giving blood samples. Research and data collection were carried out in compliance with the Helsinki Declaration, with approval of the LAU Institutional Review Board, and local ethics committees on human research.

DNA was extracted from a total of 2,968 individuals using the standard phenol-chloroform extraction procedure. Genotyping was performed on Illumina Human610 or 660W-Quad or the HumanOmniEXP-12v1 Multi-use array, as this study was cross-sectional and conducted in two phases.

SNPs underwent quality control using PLINKv1.07 (http://zzz.bwh.harvard.edu/plink/) and only individuals with a genotyping call rate of 90% were kept. A total of 2,700 subjects with an average genotyping call rate of 98.37% were used for the statistical analysis (S1 Table). After frequency pruning, SNP call rate of 95% or more and minor allele frequency (MAF) of 1% or more, 307, 238 SNPs that overlapped the different genotyping platforms remained for analysis.

The samples used in this study were previously clustered on a subset of leading principal components. There was no indication of interaction with risk factors or identified risk SNPs. The stratified populations showed no significant variations not observed in the combined population, except that the range of variability increased, consistent with smaller sample sizes [36].

### Statistical analysis

The dataset of clinical information contained 76 variables. Variables that had a high percentage of missing values (approximated 10% or more) were removed, as well as those that were deemed to be clinically unrelated to HDL-C, bringing the total number of variables to 63. Log transformed HDL-C was used as the dependent variable interest because of its skewed distribution. Automated procedures, such as forward, backward, and stepwise selection, were used to guide the process of variable selection. Ultimately, eight independent variables were chosen to be included in the final linear model, including, age, sex, diabetes status, log-transformed total cholesterol, log-transformed triglycerides, LDL cholesterol (LDL-C), BMI, and CAD category (no stenosis, stenosis ≤ 50% in 1+ vessels, stenosis > 50% in 1+ vessel). Additionally, we controlled for the genotyping platform since the first set of DNA samples (first collection) was genotyped using the Illumina Human610 or 660W-Quad while the second set of DNA samples (second collection) was genotyped using the HumanOmniEXP-12v1 Multi-use array. Smoking status was previously studied in this cohort and thus not included as a co-variate [37].

Missing values in continuous variables (total cholesterol, triglycerides, LDL-C, BMI) were imputed using their median value. Automated procedures for data with imputation and for data with the NA values removed produced similar results. Moreover, imputing NA values with the median did not drastically change the mean value of any variable (percent change between 0.05% and 1.6%). The median was chosen over the mean value as some variables had a skewed distribution and the median is resistant to outliers. By imputing rather than removing NA values, 341 subjects, that would have been otherwise removed, remained in the analysis. Imputation of clinical values was done using the R project for statistical computing (https://www.r-project.org/).

We performed a genome-wide association test that is adjusted for the following three CAD status: 1) no stenosis; 2) stenosis ≤ 50% in any of the 4 main coronary vessels, and 3) stenosis > 50% in any of the 4 main coronary vessels. Two association analyses were performed. The first, an unadjusted association analysis with each SNP as the sole predictor against log-transformed HDL-C. The second a linear model adjusted for the aforementioned, clinical variables. Both the unadjusted and adjusted linear models were additive genetic models, which measure the effect of each additional minor allele on HDL-C. All reported *p*-values from the GWA analyses are an asymptotic result based upon a Wald test. A *p*-value of 1.0x10^-8^ was used to determine genome-wide significance and a *p*-value of 1.0x10^-5^ was used for the suggestive significance cutoff.

Given that we have a quantitative phenotype, we computed the power using the QTL association feature from Purcell et al. Our sample size of 2,700 yielded a power of 15.7% while considering a total QTL variance of 0.009, based on the R^2^ of the most significant SNP, a risk allele frequency of 0.326, a D’ of 0.9, and assuming a significance level of 5E-8 [38].

## Results

The mean age of individuals was 62.33 (±11.02) years old and 72.70% of the individuals were males. The average total cholesterol level was 182.85 (±48.03) mg/dl. The average HDL-C level was 39.98 (±11.78) mg/dl. In total, 18.52% of individuals had no stenosis, 10.33% had stenosis less than or equal to 50% in at least one vessel, and 71.15% had more than 50% stenosis in at least one vessel.

The genomic inflation factor was 1.004 for the studied population. The results from the unadjusted association analysis did not find any SNPs to be significant at the genome-wide level, but did find four SNPs to be significant at the suggestive significance level (Table 2). The SNP with the smallest *p*-value from the unadjusted analysis was rs17799912 on chromosome 19 (β = 0.0524, TS = 5.023, *p* = 5.41E-07). All four SNPs had a Hardy-Weinberg chi-squared z-statistic with an absolute value less than three.

**Table 2:** Regression coefficients of significant SNPs from adjusted and unadjusted analyses.

CHR	SNP	Position in bp	Gene of Interest	Effect Allele	Beta	95% CI	*p*-value	
**Unadjusted Analysis**								
6	rs4288204	47369202	—	A	0.0366	(0.0215,0.0518)	2.29E-06	
15	rs2062091	61201424	*RORA*	C	0.0358	(0.0203,0.0513)	6.06E-06	
16	rs3764261	56993324	5' of *CETP*	T	0.0411	(0.0249,0.0572)	6.70E-07	
19	rs17799912	41535003	3' of *CYP2B6*	T	0.0524	(0.0319,0.0728)	5.41E-07	
**Adjusted Analysis**								
1	rs951307	115357015	—	T	-0.0258	(-0.0363,-0.0144)	5.56E-06	
3	rs822780	22989819	—	G	0.0239	(0.0131,0.0347)	1.54e-05	
4	rs7693827	41254862	*USHL1AS1*	T	-0.0258	(-0.0368, -0.0148)	4.19e-06	
6	rs2502465	75455385	—	T	0.0298	(0.0169,0.0426)	5.61e-06	
8	rs7835508	38782627	*PLEKHA2*	C	-0.0273	(-0.0393,-0.0153)	8.21e-06	
12	rs1599780	63834115	5' of *DPY19L2*	A	-0.0682	(-0.0978,-0.0387)	6.05e-06	
15	rs934297	58752734	*LIPC*	A	-0.0252	(-0.0360, -0.0144)	4.84e-06	
**16**	**rs3764261**	**56993324**	5' of *CETP*	**T**	** -0.0325**	**(-0.0438,-0.0212)**	**1.86e-08**	**
16	rs4783961	56994894	5' of *CETP*	A	-0.0233	(-0.0341, -0.0126)	1.92e-05	
16	rs1800775	56995236	5' of *CETP*	C	0.0256	(0.0150, 0.0362)	2.11e-06	

** *P*-value reaching genome-wide significance

**Fig 1:**
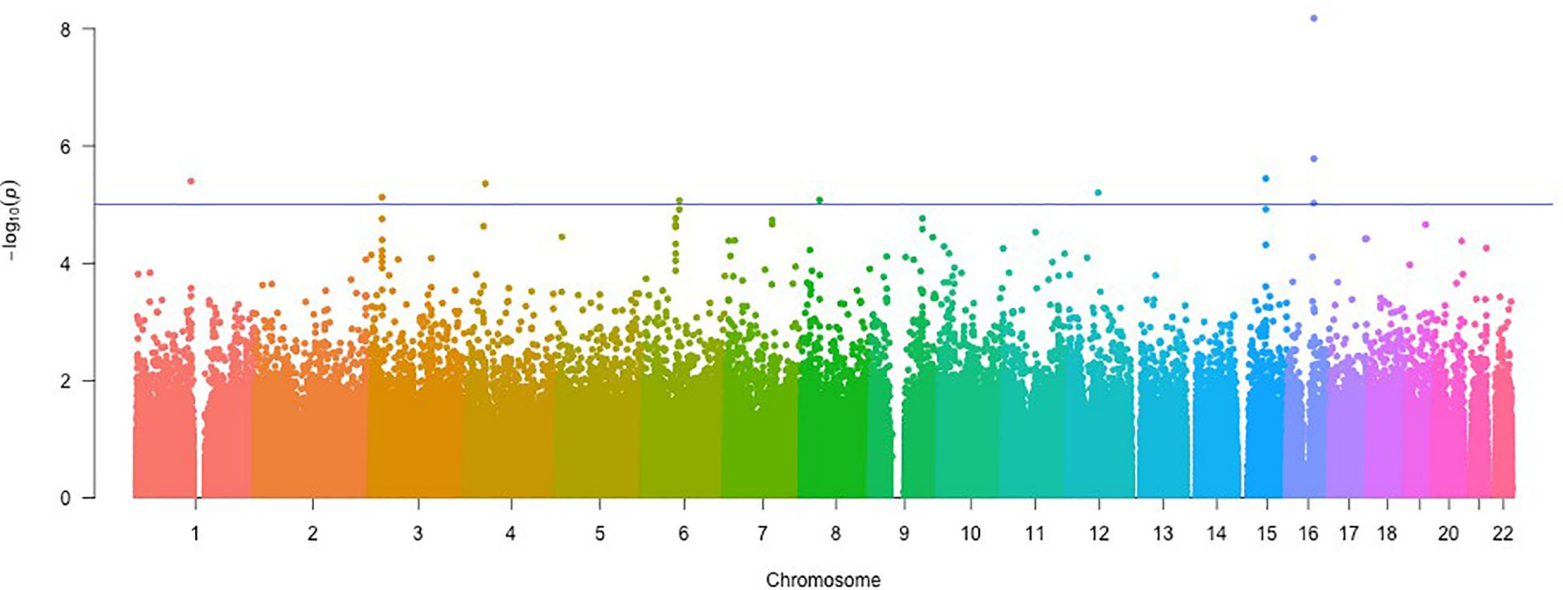
Manhattan plot of results from adjusted GWA analysis: *P*-values reported are from the model adjusted for biological variables. Y-axis is shows the significance (-log_10_ p-value) of each association. X-axis is split by chromosome and ordered by position in base pairs. A total of 307,238 SNPs were tested in an association analysis of 2,700 Lebanese individuals. Line at suggestive significance cutoff (1x10^-5^).

**Fig 2:**
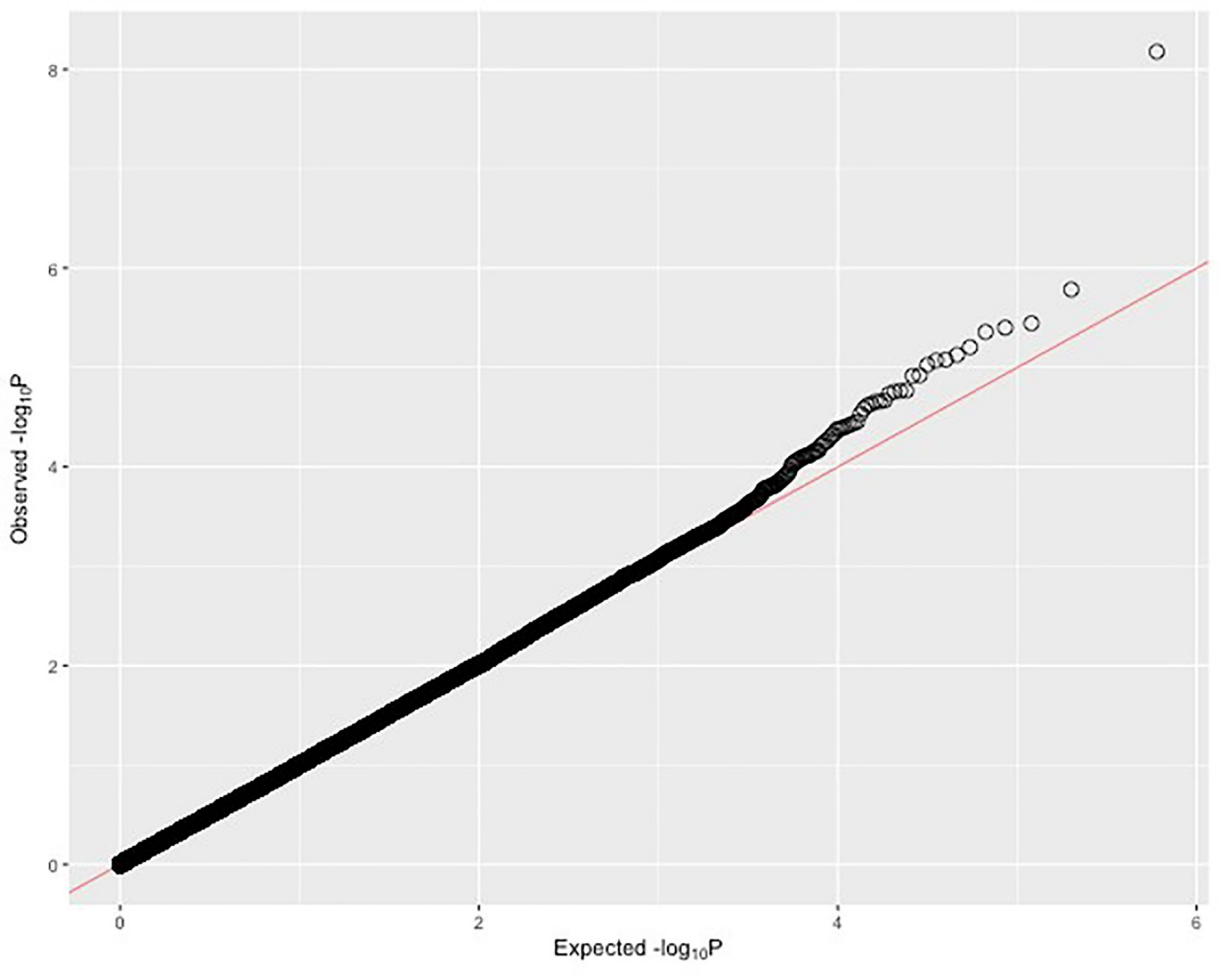
Q-Q plot of results from adjusted GWA analysis: For each point, y-axis is the observed–log_10_ *p*-value of association from a model adjusted for biological variables. X-axis is the expected–log_10_ *p*-value under the null hypothesis. The line represents same expected and observed *p*-value for every SNP. Deviations above the line show observed *p*-values higher than expected.

The adjusted analysis yielded one SNP that reached genome-wide significance while adjusting for age, sex, diabetes status, total cholesterol, triglycerides, LDL-C, BMI, CAD category, (Fig 1, Table 3). The SNP with genome-wide significance, rs3764261, is on chromosome 16 (β = -0.03239, *p* = 2.38E-8). The MAF of rs3764261 is 0.326. There is no evidence of systematic over-dispersion of the test statistics, intrinsic bias, or genotyping error in the results of the adjusted analysis (Fig 2). This SNP showed borderline genome-wide significance in the unadjusted analysis (*p* = 6.695E-7, Table 2). Nine other SNPs were found to be above the suggestive significance threshold (Table 2). Two of these SNPs are on chromosome 16, and in close proximity, 1.57 and 1.91 Kbp, to rs3764261. These SNPs are respectively rs4783961 (β = 0.02428, TS = 4.439, *p* = 9.420E-06) and rs1800775 (β = -0.02598, TS = -4.803, *p* = 1.647E-06). The MAF of these two SNPs were 0.490 and 0.465, respectively. The LD between the top SNP rs3764261 is found to be R^2^ = 0.436; D’ = 0.931 with rs4783961 and R^2^ = 0.418; D’ = 0.995 for rs1800775. All significant SNPs had a Hardy-Weinberg chi-squared z-statistic with an absolute value less than three.

**Table 3:** Regression coefficients from adjusted model rs3764261.

Variable	ß^	Test Statistic	*p*-value
rs3764261	-0.03255	-5.641	<0.0001
Age	0.00123	3.22	0.001295
Sex (F)	0.1056	11.405	<0.0001
Diabetes	0.00401	0.457	0.647
log (total cholesterol)	1.2342	36.066	<0.0001
log (triglycerides)	-0.32419	-37.116	<0.0001
LDL-C	-0.004452	-21.743	<0.0001
BMI	-0.00284	-3.089	0.00203
CAD Category			
No Stenosis	Ref	—	—
≤ 50% Stenosis in 1+ Vessels	0.02351	1.543	0.1230
> 50% Stenosis in 1+ Vessels	-0.00776	-0.706	0.4800
HumanOmniExp-12v1	-0.04294	-3.961	<0.0001

With corresponding t-test statistic and *p*-value.

## Discussion

In this study, we examined genetic variants to determine alleles associated with HDL-C levels, due to the role of HDL-C in the underlying pathology of disease such as CAD. Using genome-wide association analysis techniques, we identified several SNPs associated with HDL-C levels in the Lebanese population using unadjusted and adjusted by biological factors models. This study reports the first genome-wide analysis of HDL-C in the Lebanese, let alone Middle Eastern, population. The performance of genome-wide association studies across a variety of ethnicities is important to refine our understanding of complex human diseases. Such inclusion of diverse ethnicities and geographically distant populations in conjunction with the use of linkage analysis and well as admixture analysis allow for better determination of the genomic location of causal mutations [33, 39].

This study found no SNPs to be significant at the genome-wide level in the unadjusted model and one SNP to be significant at the genome-wide level in the adjusted model. This SNP, rs3764261, lies 5’ of *CETP* on chromosome 16q13 and has been previously found to be associated with HDL-C levels across a wide array of populations, including European and Japanese populations (S2 Table) [31, 40–43]. Moreover, this is the only SNP to be found at the suggestive level (*p* = 6.695E-07) in the unadjusted analysis and crossing the threshold for genome-wide significance in the adjusted analysis (*p* = 6.653E-09). The result of the adjusted analysis shows that HDL-C level increases by 3.41% for every additional minor allele, T, while adjusting for biological variables in the model. The average HDL-C levels for genotypes GG, GT, and TT, are 38.9, 40.5, 42.5 mg/dl, respectively. The two additional SNPs found to be significant at the suggestive level on chromosome 16 in the adjusted analysis are located in the 5’ region of the CETP locus. The first, rs4783961, showed a similar effect, such that for every additional minor allele, A, HDL-C level increases by 2.46%, while adjusting for all other variables in the model. The average HDL-C levels for genotypes GG, GA, and AA, are 38.7, 40.3, 40.6 mg/dl, respectively. By contrast, rs1800775, finds a lowering effect for each additional minor allele, C, such that for every additional minor allele HDL-C level decreases by 2.56%, while adjusting for all other variables in the model. The average HDL-C levels for genotypes AA, AC, and CC, are 41.3, 39.8, 38.5mg/dl, respectively. All reported SNPs were compared to previously performed GWAS through https://www.ebi.ac.uk/gwas/, as well as the Global Lipids Genetics Consortium database (S2 Table). Two SNPs were previously reported to be associated with levels of HDL-C, rs3764261 (p-value: 1.39E-769and rs1800775 (p-value = 3.33E-644). [44–46]. The other SNPs found significant at the suggestive level have not been reported to date in other studies. Interestingly, rs934297 of the hepatic lipase gene, *LIPC*, has been found to be associated at a suggestive level.

While it makes great strides in identifying disease-associated loci, even going so far as to support earlier findings from other ethnicities, there are some limitations to this study. Decreased HDL cholesterol levels have constantly been associated with coronary artery disease and type II diabetes, which may explain that we did not find variants with genome-wide levels of statistical significance in our cohort. A number of individuals in this study were taking lipid lowering medications, which are likely to alter their plasma lipid values, specifically LDL-C, profile. Given the low power of this study to identify loci associated with HDL-C at GWAS level, some loci previously described to be associated with HDL-C, such as CYP7A1, NPC1L1 and SCARB1, did not reach genome wide significance level in our cohort. Replication analysis, with an increase in the number of enrolled participants would help unravel the role of more rare variants in the etiology of cardiovascular diseases in relation to HDL-C [47]. It may also be beneficial to investigate the use of imputation of additional SNPs based on their linkage with already genotyped SNPs as this could lead to the discovery of additional significant SNPs at the genome-wide level. The use of linkage between SNPs will help to fine map the exact location of causal SNPs. Additionally, the common disease/common variant hypothesis states that common disorders, such as low HDL-C or CAD, are likely influenced by genetic variants that have a high minor allele frequency. If the genetic variants that effect common diseases have a high MAF, as the three found to be significant in *CETP* did, then they are likely to have a small effect size, such that each individual SNP accounts for a relatively small amount of the variation in the common disease. In turn, if a common disorder shows heritability, as HDL-C does, then many common genetic variants must account for the heritable genetic risk [32, 48]. For such reasons, future studies should include haplotypes to determine if a specific sequence of alleles is associated with and account for a larger portion of the variation in HDL-C levels in the population. HDL-C levels are clinically used on a daily basis in cardiovascular risk assessment tools, and an understanding of their variation is core to the understanding and treatment of cardiovascular diseases. Continued exploration of genetic risk loci of HDL-C is fundamental to an understanding of such diseases.

## Supporting information

S1 Table Studied population. For the 2,700 individuals who passed the QC, 2,171 individuals were genotyped using the Illumina Human610/660W chips and 529 were genotyped using the HumanOmniExp-12v1 chip.
(XLSX)

S2 Table Major published association results on rs1800775 and rs3764261 in different populations. RAF, risk allele frequency.
(XLSX)

S1 File Supplementary data. All data underlying the findings described in the manuscript.
(XLSX)
