# Correction: Genome-wide association analysis of HDL-C in a Lebanese cohort

**DOI:** 10.1371/journal.pone.0221672

**Published:** 2019-08-22

**Authors:** Rebecca Deek, Jason Nasser, Anthony Ghanem, Marc Mardelli, Georges Khazen, Angelique K. Salloum, Antoine Abchee, Michella Ghassibe-Sabbagh, Pierre Zalloua

One of the affiliations for the ninth author is incorrect. Pierre Zalloua is not affiliated with #3 but with #2: School of Medicine, Lebanese American University, Beirut, Lebanon.
